# Population Structure and Eigenanalysis

**DOI:** 10.1371/journal.pgen.0020190

**Published:** 2006-12-22

**Authors:** Nick Patterson, Alkes L Price, David Reich

**Affiliations:** 1 Broad Institute of Harvard and MIT, Cambridge, Massachusetts, United States of America; 2 Department of Genetics, Harvard Medical School, Boston, Massachusetts, United States of America; University of Alabama at Birmingham, United States of America

## Abstract

Current methods for inferring population structure from genetic data do not provide formal significance tests for population differentiation. We discuss an approach to studying population structure (principal components analysis) that was first applied to genetic data by Cavalli-Sforza and colleagues. We place the method on a solid statistical footing, using results from modern statistics to develop formal significance tests. We also uncover a general “phase change” phenomenon about the ability to detect structure in genetic data, which emerges from the statistical theory we use, and has an important implication for the ability to discover structure in genetic data: for a fixed but large dataset size, divergence between two populations (as measured, for example, by a statistic like *F_ST_*) below a threshold is essentially undetectable, but a little above threshold, detection will be easy. This means that we can predict the dataset size needed to detect structure.

## Synopsis

When analyzing genetic data, one often wishes to determine if the samples are from a population that has *structure*. Can the samples be regarded as randomly chosen from a homogeneous population, or does the data imply that the population is not genetically homogeneous? Patterson, Price, and Reich show that an old method (principal components) together with modern statistics (Tracy–Widom theory) can be combined to yield a fast and effective answer to this question. The technique is simple and practical on the largest datasets, and can be applied both to genetic markers that are biallelic and to markers that are highly polymorphic such as microsatellites. The theory also allows the authors to estimate the data size needed to detect structure if their samples are in fact from two populations that have a given, but small level of differentiation.

## Introduction

A central challenge in analyzing any genetic dataset is to explore whether there is any evidence that the samples in the data are from a population that is structured. Are the individuals from a homogeneous population or from a population containing subgroups that are genetically distinct? Can we find evidence for substructure in the data, and quantify it?

This question of detecting and quantifying structure arises in medical genetics, for instance, in case-control studies where uncorrected population structure can induce false positives [1]. It also arises in population genetics, where understanding of the structure may be important to the key scientific issues, especially uncovering the demographic history of the population under study.

We focus on principal components analysis (PCA), which was first introduced to the study of genetic data almost thirty years ago by Cavalli-Sforza [2].

We will use PCA and “eigenanalysis” interchangeably. The latter term focuses attention on the fact that not just the eigenvectors (principal components) are important here, but also the eigenvalues, which underlie our statistical procedures.

PCA has become a standard tool in genetics. In population genetics, we recommend a review paper [3] focusing on the use of “synthetic maps” which use PCA to study genetic geographic variation.

Usually PCA been applied to data at a population level, not to individuals as we do here. Exceptions are [4,5].

In addition to single nucleotide polymorphisms (SNPs) and microsatellites, PCA has been applied to haplotype frequencies [6,7] and the distribution of ALU insertion polymorphisms [8] in order to study population structure. Most of the literature on PCA in genetics is applied, not methodological, and we know of no paper that concentrates as we do here on the statistical significance of the components. Data with hundreds or thousands of individuals and hundreds of thousands of markers are now becoming available, so that small but real effects will be detectable, and it is important to develop rigorous tests for population structure that will be practical, even on the largest datasets. This is our main aim in this paper.

Using some recent results in theoretical statistics, we introduce a formal test statistic for population structure. We also discuss testing for *additional* structure after some structure has been found. Finally, we are able to estimate the degree of population differentiation that will be detectable for a given data size.

Our methods work in a broad range of contexts, and can be modified to work with markers in linkage disequilibrium (LD). The methods are also able to find structure in admixed populations such as African Americans—that is, in which individuals inherit ancestry from multiple ancestral populations—as long as the individuals being studied have different proportional contributions from the ancestral populations.

We believe that principal components methods largely fell out of favor with the introduction of the sophisticated cluster-based program STRUCTURE [9,10]. STRUCTURE and similar methods are based on an interpretable population genetics model, whereas principal components seems like a “black box” method. We will discuss how the models underlying the cluster methods, and the PCA technique we will describe, are much closer to each other than they may at first appear to be.

Our implementation of PCA has three major features. 1) It runs extremely quickly on large datasets (within a few hours on datasets with hundreds of thousands of markers and thousands of samples), whereas methods such as STRUCTURE can be impractical. This makes it possible to extract the powerful information about population structure that we will show is present in large datasets. 2) Our PCA framework provides the first formal tests for the presence of population structure in genetic data. 3) The PCA method does not attempt to classify all individuals into discrete populations or linear combinations of populations, which may not always be the correct model for population history. Instead, PCA outputs each individual's coordinates along axes of variation. An algorithm could in principle be used as a post-processing step to cluster individuals based on their coordinates along these axes, but we have not implemented this.

We note that STRUCTURE is a complex program and has numerous options that add power and flexibility, many of which we cannot match with a PCA approach. Perhaps the central goal of STRUCTURE is to classify individuals into discrete populations, but this is not an object of our method. We think that in the future both cluster-based methods such as STRUCTURE and our PCA methods will have a role in discovering population structure on genetic data, so that, for example, our PCA methods offer a good default for the number of clusters to use in STRUCTURE. In complex situations, such as uncovering structure in populations where all individuals are equal mixtures of ancestral populations, it may remain necessary to use statistical software that explicitly models admixture LD, such as [10–13], which allow estimation of local ancestry at arbitrary points of the genome.

In this study we aim to place PCA as applied to genetic data on a solid statistical footing. We develop a technique to test whether eigenvectors from the analysis are reflecting real structure in the data or are more probably merely noise. Other papers will explore applications to medical genetics [14] and to the uncovering of demographic history. In this paper, our main purpose is to describe and to validate the method, rather than to make novel inferences based on application to real data, which we leave to future work. We show that statistically significant structure is real and interpretable, and also that our methods are not failing to recover real structure that is found by other techniques.

Two important results emerge from this study. First, we show that application of PCA to genetic data is statistically appropriate, and provide a formal set of statistical tests for population structure. Second, we describe a “phase change” phenomenon about the ability to detect structure that emerges from our analysis: for a fixed dataset size, divergence between two populations (as measured, for example, by a statistic like *F_ST_*) that is below a threshold is essentially undetectable, but a little above threshold detection will be easy. Based on these results, we are able to give an estimate of how much data will be required to find population structure given a level of genetic divergence such as *F_ST_* (as defined by Cavalli-Sforza, [15, p. 26, Equation 3].)

The theory shows that the methods are sensitive, so that on large datasets, population structure will often be detectable. Moreover, the novel result on the phase change is not limited just to PCA, but turns out to reflect a deep property about the ability to discover structure in genetic data. For example, in the paper we present simulations that show the ability to detect structure occurs with the same dataset size when STRUCTURE and PCA are used; that is, the phase change manifests itself in the same place.

The phase change effect was suggested by a recent paper in theoretical statistics [16], which demonstrated the phenomenon for a situation that is mathematically similar to ours. The theory has continued to develop and nearly all we need has now been proved, the most recent paper being [17]. We believe that the applications to genetics still pose some interesting questions for the theorists. While our methods are derived from asymptotic theory (where the datasets are very large), they also seem to work well on small datasets, and we would be interested in seeing a theoretical explanation.

## Results

The basic technique is simple. We assume our markers are biallelic, for example, biallelic single nucleotide polymorphisms (SNPs). Regard the data as a large rectangular matrix *C,* with rows indexed by individuals, and columns indexed by polymorphic markers. For each marker choose a reference and variant allele. We suppose we have *n* such markers and *m* individuals. Let *C*(*i,j*) be the number of variant alleles for marker *j*, individual *i.* (Thus for autosomal data we have *C*(*i,j*) is 0,1 or 2.) For now suppose that there is no missing data. From each column we subtract the column means. So set for column *j*:

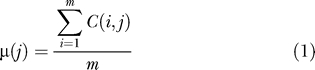
and then the corrected entries are:

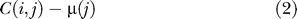



Set *p*(*j*) = *μ*(*j*)/2, an estimate of the underlying allele frequency (autosomal data). Then each entry in the resulting matrix is

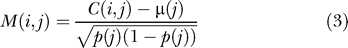

Equation 3 is a normalization step, which is motivated by the fact that the frequency change of a SNP due to genetic drift occurs at a rate proportional to 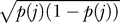 per generation. It also normalizes (at least if the data is in Hardy-Weinberg equilibrium) each data column to have the same variance. We note that Nicholson et al. use the same normalization, and motivate it similarly [18].

We verified (unpublished data) that the normalization improves results when using simulated genetic data, and that on real data known structure becomes clearer. (However all the results are just as mathematically valid even without the normalizations.)

The methods also are applicable to data such as microsatellites, where there are more than two alleles at a single site. We use a device of Cavalli-Sforza [2,15], making a “marker” *j* out of each allele, and then setting *C*(*i*,*j*) to be the number of occurrences of the allele for sample *i*. We omit the normalization step of Equation 3 for microsatellites, merely subtracting the mean. The normalization has no clear justification for microsatellite data, and results on real data (unpublished) show that it produces worse performance in this case.

An alternative, suggested by a referee, is to use the microsatellite allele length as a continuous variable, and carry out PCA directly after a suitable normalization.

Now we carry out a singular value decomposition on the matrix *M*. (A standard reference for the numerical methods is [19]. Public domain software is readily available—we used the well-known package LAPACK, http://www.netlib.org/lapack.) We are chiefly interested here in the case that the number of samples is less than the number of markers: *m* < *n*. Computationally we will form

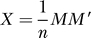
the sample covariance of the columns of *M*. The resulting matrix is *m* × *m*, with a dimension equal to the number of samples in the dataset. We then compute an eigenvector decomposition of *X*. Eigenvectors corresponding to “large” eigenvalues are exposing nonrandom population structure. This means that a central issue for this paper is what is “large” here, or, more precisely, what is the distribution of the largest eigenvalues of *X* at random (when there is no population structure)?

The method is fast. In practice, the running time is dominated by the calculation of the matrix product *MM′,* which for extremely large problems is readily computed on a parallel architecture. On a fast workstation, the matrix product for a dataset of 100 individuals and 100,000 markers takes just four seconds. For data with *m* individuals and *n* markers, the work is proportional to *m* ^2^ *n,* and thus for a set of 2,000 individuals and 500,000 markers would take about 2.5 hours on the same single processor (see Methods for more details). For many of the problems we have analyzed, reading and storing the data takes longer than the analysis.

Most, though not all, previous applications of PCA to analysis of population structure have taken the data to be a matrix where the rows are indexed by populations not by individuals (e.g., [2,15]). We prefer to consider the larger array where the rows are indexed by individuals. Even when we have population labels, it is useful to examine within-population variation, and we also are often able to find outliers in the data. Furthermore, when population labels are available, we can carry out an analysis to check that the labels do correspond to structure that the eigenanalysis has uncovered.

We note that population labels may be socially constructed. This makes us nervous about employing them in an initial data study. On the other hand, the individual samples certainly do not have any such construct, and even if population labels are available, initial analysis at an individual level allows us to check the meaningfulness of the labels [20].

Cavalli-Sforza [15, pp. 39–42] gives an explanation of why PCA can be expected to reveal population structure. We give a different explanation, oriented towards analysis at the individual level. If **e** ^[1]^ is the principal eigenvector of the matrix *X,* this means that the sum of squares

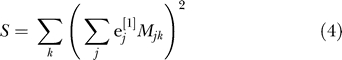
is maximized over all vectors with constant norm. The second eigenvector **e** ^[2]^ maximizes the same expression with the constraint that **e** ^[1]^, **e** ^[2]^ are orthogonal, and so on. Why would we expect this to reveal population structure? Suppose that in our sample, we have just two populations and that each is homogeneous. Choose a vector with coordinates constant and positive for samples from one population, and coordinates constant and negative for samples from the other. Arrange so that the vector coordinates sum to zero. Then, since alleles within a population will tend to agree more than in the sample as a whole, the quantity *S* of Equation 4 will tend to be large. This is exactly what we maximize as a function of the vector **e**. More generally, if we have *K* distinct populations, there are *K* − 1 vectors constant on each population, summing to zero and linearly independent. This implies that, if the number of markers is sufficiently large, there will be *K* − 1 eigenvalues and *K* − 1 corresponding eigenvectors of our matrix that are significant and meaningful. Vectors orthogonal to these *K* − 1 vectors are showing within-population variance, and if each population is homogeneous, this is just reflecting sampling noise.

### Tracy–Widom Theory

We now turn to some recent theoretical statistics. Consider an *m* × *n* matrix *M*, each entry of which has an independent standard normal random variable. We are interested in the case that *m* < *n*.

(A notational issue is that in our genetic data, if *m* is the number of individuals, then the square matrix for which we calculate eigenvalues has rank *m* − 1 [we lose a dimension by forcing each column to sum to zero]. We will, for the majority of the paper, write *m*′ = *m* − 1 for the number of nonzero eigenvalues. However in this theoretical section, we will assume there are *m* nonzero eigenvalues.)

Let

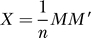



*X* is a *Wishart* matrix. Let {*λ_i_*}_1__≤*i*≤*m*_ be the eigenvalues of *X*. The probability density of (*λ*_1_,… *λ_m_*) is known [21] but not directly relevant to our work here, so we omit the details.

Order the eigenvalues so that

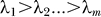



Johnstone in a key paper [22] showed that suitably normalized, and for *m, n,* large, the distribution of the largest eigenvalue *λ*_1_ is approximately a distribution discovered by Tracy and Widom [23], which in this paper we call TW. Set

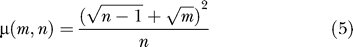


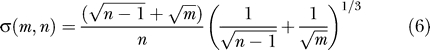
Now set

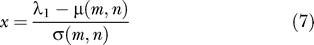
Then the distribution of *x* is approximately TW.

More precisely, if as *m*,*n* → ∞, *n*/*m* → *γ* ≥ 1, then Johnstone proves [22, Theorem 1.1] that *x* tends to TW in distribution. As we show later (Theorem 2), Johnstone's theorem also holds if in the expression for *x* in Equation 7, we replace *x* by:

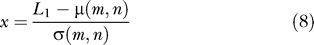
where

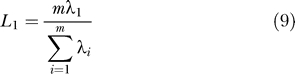



The only difference here is that the *m* eigenvalues have been normalized to have sum *m*.

In [22] Johnstone proved his theorem for the case that *n*,*m* → ∞ with *m*/*n* bounded away from 0, but this condition was shown in [23] not to be necessary. Johnstone [22] gives convincing evidence that the fit is good even for values as small as *m* = 5, *n* = 20.

We show in Figure 1 a plot of the Tracy–Widom density.

**Figure 1:**
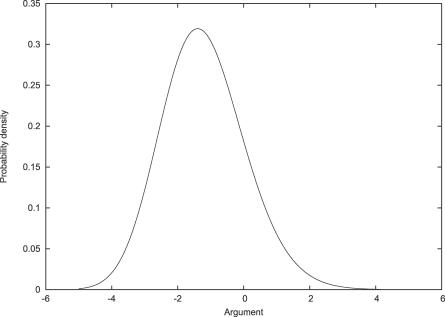
The Tracy–Widom Density Conventional percentile points are: *P* = 0.05, *x* = .9794; *P* = 0.01, *x* = 2.0236; *P* = 0.001, *x* = 3.2730.

The complexity of the TW definition is irrelevant to its application to real data. One computes a statistic, and then looks up a *p*-value in tables or through a computational interface. This is little different from how one uses (say) a conventional chi-squared test.

One concern with applying this approach to genetic data is that the entries in the matrix *M* do not have the Gaussian distributions expected for a Wishart matrix; instead, they correspond to the three possible genotypes at each SNP. However it is not critical that the entries in the *m* × *n* matrix *M* be Gaussian. Soshnikov [25] showed that the same TW limit arose if the cell entries were any distribution with high-order moments no greater than the Gaussian. The matrix *X* is a sum of *n* rank 1 matrices, and Soshnikov's result suggests that the same limit would be obtained from any probability distribution in which the columns of *M* are independent, isotropic (all directions are equiprobable), and such that the column norms have moments no larger than those for a column of independent Gaussian entries. In all our genetic applications, the column norms are in fact bounded, so we can expect the sample covariance matrices to behave well.

This theory, originally developed for the case of Gaussian matrix entries, thus seemed likely to work well with large genetic biallelic data arrays. The remainder of this paper verifies that this is the case.

### Applying Eigenanalysis to Datasets with Linked Markers

For genetic applications we cannot necessarily assume that all our markers are unlinked and thus independent. For instance, in the International Haplotype Map project [26], markers were chosen about 5,000 bases apart (phase 1), or about 1,000 bases apart (phase 2), and so nearby markers will often be in LD. Mathematically this will induce correlation between nearby columns of our matrix *M*. The effect of this will be that the matrix

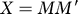
should be “Wishart-like,” but the nonindependence of the columns will reduce the effective sample size. We will discuss this further (see Correcting for LD) but now introduce a new idea. This adds robustness to our methods, so that minor deviations from the model become of lesser importance.

Suppose we have *m* individuals. We will analyze *X* = *MM′* as a Wishart matrix. The rank of *X* will be *m* − 1 (assuming we have many SNPs compared with *m*). We will think of the eigenvalues of *X* as coming from a (*m* − 1) × (*m* − 1) Wishart, and write *m*′ = *m* − 1.

There are two unknowns that we can regard as parameters to the Wishart: 1) *σ* ^2^: the variance of the normal distribution used for the cells of the rectangular matrix *M*; 2) *n*′: The number of columns of *M*.

We want to carefully distinguish here between *n,* the *actual* number of columns of our data array, and *n′,* a theoretical statistical parameter, modeling the approximate Wishart distribution of the square matrix *X*. We originally fit *σ*,*n′* by maximum likelihood. The likelihood, as a function of the two parameters, has two sufficient statistics, which are 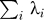 , and 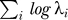 . Maximum likelihood did not always work well, in our genetic applications, probably because 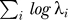 is sensitive to *small* eigenvalues, while we are only interested in large. We recommend a moments estimator:

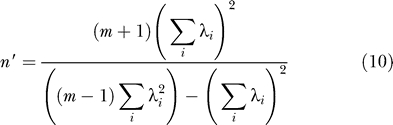
which is justified later. Note that *n′* is invariant to scaling of the matrix *M* as it should be. We estimate *σ* by:

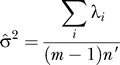



### A Test for Population Structure

This leads immediately to a formal test for the presence of population structure in a biallelic dataset.

1. Compute the matrix *M* as in Equations 1, 2 and 3. *M* has *m* rows, *n* columns.

2. Compute *X* = *MM′*. *X* is *m* × *m*.

3. Order the eigenvalues of *X* so that

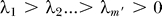
where *m′* = *m* − 1. (On a large dataset *X* will always have rank *m′*.)

4. Using the eigenvalues *λ_i_* (1 ≤ *i* ≤ *m*′), estimate *n*′ from Equation 10.

5. The largest eigenvalue of *M* is *λ*_1_. Set

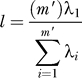



6. Normalize *l* with Equations 5–7, where the effective number of markers *n*′ replaces *n*. This yields a test statistic *x* = *x*(*M*).

Our statistic *x*(*M*) is approximately TW-distributed. A *p*-value can now be computed from tables of the TW distribution. Notice that our statistic is independent of the scaling of the *λ_i_,* and it is convenient to normalize by scaling so that the sum of the eigenvalues (that is the trace of *M*) is *m*′. All eigenvalues we report are scaled in this manner.

### Simulations to Demonstrate Robustness of the Tests of Significant Structure

We first made a series of simulations in the absence of population structure. (Some additional details are in the Methods section.) Our first set of runs had 100 individuals and 5,000 unlinked SNPs, and the second 200 individuals and 50,000 unlinked SNPs. In each case we ran 1,000 simulations and show in Figure 2A and 2B probability–probability (P–P) plots of the empirical and TW tail areas. The results seem entirely satisfactory, especially for low *p*-values in the top right of Figures 2A and 2B. For assessment of statistical significance, it is the low *p*-value range that is relevant. The simulations show more generally that the TW theory is relevant in a genetic context, that the normalizations of Equations 5–7 are appropriate, and that the calculation of the effective marker size has not seriously distorted the TW statistic.

**Figure 2:**
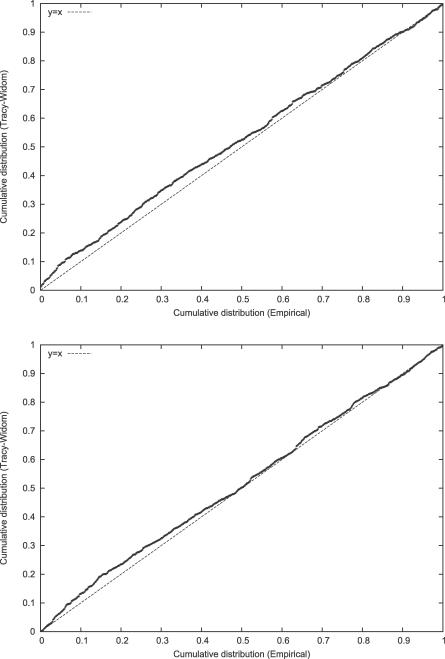
Testing the Fit of the TW Distribution (A) We carried out 1,000 simulations of a panmictic population, where we have a sample size of *m* = 100 and *n* = 5,000 unlinked markers. We give a P–P plot of the TW statistic against the theoretical distribution; this shows the empirical cumulative distribution against the theoretical cumulative distribution for a given quantile. If the fit is good, we expect the plot will lie along the line *y* = *x*. Interest is primarily at the top right, corresponding to low *p*-values. (B) P–P plot corresponding to a sample size of *m* = 200 and *n* = 50,000 markers. The fit is again excellent, demonstrating the appropriateness of the Johnstone normalization.

### Detecting Additional Structure

It is very important to be able to answer the question: *Does the data show evidence of additional population structure over and above what has already been detected?* The test we propose is extremely simple. If our matrix *X* has eigenvalues

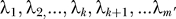
and we already have declared the top *k* eigenvalues to be significant, then we simply test λ*_k_*_+1_,…λ*_m_* as though *X* was a (*m*′ − *k*) × (*m*′ − *k*) Wishart matrix. Johnstone shows [22, Proposition 1.2] that this procedure is conservative, at least for a true Wishart matrix. We tested this by generating data in which there is one eigenvalue that is overwhelmingly significant, and examined the distribution of the *second* eigenvalue. As shown by the P–P plot of Figure 3, the fit is again very good, especially for small *p*-values. If an eigenvalue is not significant, then further testing of smaller eigenvalues should not be done.

**Figure 3:**
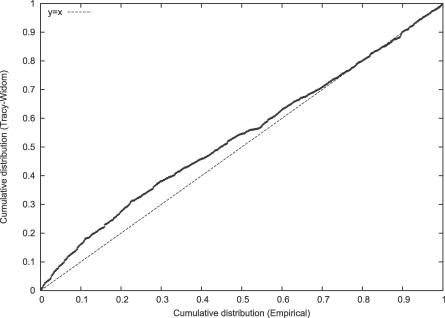
Testing the Fit of the Second Eigenvalue We generated genotype data in which the leading eigenvalue is overwhelmingly significant (*F_ST_* = .01, *m* = 100, *n* = 5,000) with two equal-sized subpopulations. We show P–P plots for the TW statistic computed from the *second* eigenvalue. The fit at the high end is excellent.

### Cluster Analysis and PCA

There is a much closer relationship between our PCA and a cluster-based analysis than is at first apparent. Consider a model of genetic structure where there are *K* populations, and fix a marker and variant allele. The populations have diverged from an ancestral population recently. Suppose that the allele frequency of the variant in the ancestral population is *P,* and in population *i* is *p_i_*. Conditional on *P,* assume that **p** = (*p*_1_,*p*_2_,…*p_K_*) has mean (*P*,*P*,…*P*) and covariance matrix *P*(1 − *P*)*B* for some matrix *B*. Much past work in genetics uses this paradigm, with variations on the distribution of *B,* and on the detailed distribution of **p** conditional on *P;* for instance, both Nicholson et al. [18] and STRUCTURE [9] in “correlated frequency mode,” and in the “F-model” of [10]. The setup here is nearly inevitable if one is considering allele frequencies in populations that have only diverged a small amount. In [18, p. 700] for the case of a diagonal matrix *B,* it is shown that the diagonal term *B_ii_* can be interpreted as the “time on the diffusion time-scale” (inversely proportional to effective population size) in which population *i* has undergone genetic drift.

Suppose we sample (autosomal) genotypes from these *K* populations. Assume there are *M_i_* samples from population *i,* and set

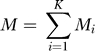
We suppose that the divergence of each population from a root population, as measured by *F_ST_* or equivalently by divergence time on the diffusion timescale, is of order *τ,* which is small. What are the eigenvalues of the theoretical covariance *C* of the samples for the marker after our mean adjustment and normalization? Let *M* become large, while the relative abundance of the samples stays constant across populations. It can be shown (see the mathematical details, Theorem 3) that if *B* has full rank, then *C* has *K* − 1 large eigenvalues that tend to infinity with *M, M* − *K* eigenvalues that are 1 + *O*(*τ*) and one zero eigenvalue that is a structural zero, arising from the fact that our mean adjusted columns all have zero sum. We are interested in the case that *τ* ≪ 1 while *M* ≫ 1.

Thus, natural models of population structure predict that most of the eigenvalues of the theoretical covariance will be “small,” nearly equal, and arise from sampling noise, while just a few eigenvalues will be “large,” reflecting past demographic events. This is exactly the situation that Johnstone's application of Tracy–Widom theory addresses. We also note that on real data (as we show below), the TW theory works extremely well, which shows that the model will be a reasonable approximation to “truth” in many cases.

### Axes of Variation

Thus, we expect that the theoretical covariance matrix (approximated by the sample covariance) will have *K* − 1 “large” eigenvalues, with the remainder small and reflecting the sampling variance. We call the eigenvectors of the theoretical covariance, corresponding to the large eigenvalues, “axes of variation.” These are a theoretical construct, as of course we only observe the sample covariance. Nevertheless, for eigenvectors that are highly significant by our tests, we expect the corresponding eigenvector to correlate well with the true “axis of variation.” An analogy here is that we would often think of an allele as having a “true” population frequency in a population, and would regard the frequency of the allele in a sample as an estimator of the true frequency.

As defined, our axes of variation do depend on the relative sample sizes of the underlying populations, so that differences between populations each with a large sample size are upweighted. This is worth remembering when interpreting the results, but does not seem a major impediment to analysis.

### A Formal Test Is Appropriate in Our Applications

We do not review in detail older methods for testing for significance. One technique is the “broken stick” model [27,28], used, for instance, in a recent population genetics analysis [5]. In this model, one normalizes the *m*′ nonzero eigenvalues to sum to 1, then sorts them in decreasing order, and compares the *k*-th eigenvalue with the expected size of the *k*-th largest subinterval of the unit interval, partitioned by “breaking” the interval at *m*′ − 1 uniformly chosen points. This method does not use the number of markers in any way, thus it cannot be making optimum use of the data. In particular for datasets with large numbers of markers, real population structure may go undetected.

We believe that the application of PCA to genetic data—and our way of analyzing the data—provides a natural method of uncovering population structure, for reasons that are subtle; thus, it is useful to spell them out explicitly. In most applications of PCA, the multivariate data has an unknown covariance, and PCA is attempting to choose a subspace on which to project the data that captures most of the relevant information. In many such applications, a formal test for whether the true covariance is the identity matrix makes little sense.

In genetics applications we believe the situation is different. Under standard population genetics assumptions such as a panmictic population, the natural null is that the eigenvalues of the true covariance matrix are equal, a formal test is appropriate, and deviations from the null are likely to be of real scientific and practical significance. To support this, in our experience on real data we take our null very seriously and attempt to explain all statistically significant axes of variation. Often the explanation is true population structure in the data, but we also often expose errors or difficulties in the data processing. Two examples follow.

In some population genetic data from African populations, the fourth axis of variation showed some San individuals at each end of the axis. This made little genetic sense, and the cause was some related samples that should have been removed from the analysis.

In a medical genetic study, an unexplained axis of variation was statistically correlated with the micro-titer plates on which the genotyping had been carried out. This suggested that the experimental setup was contributing to the evidence for structure, instead of real population differentiation.

In both these cases more careful data preprocessing would have eliminated the problem, but analysis and preparation of large datasets is difficult, and more tools for analysis and error-detection are always of benefit.

### ANOVA Statistics Given Labeled Populations

In many practical applications, samples will already be grouped into subpopulations (for instance, in medical genetics there are often two populations: cases and controls). It is natural to want to test if our recovered eigenvectors reflect differences among the labeled subpopulations. We therefore fix some eigenvector, and can regard each individual as associated with the corresponding coordinate of the eigenvector. We want to test if the means of these coordinate values in each subpopulation differ significantly. Our motivation is firstly that this is a powerful check on the validity of our (unsupervised) Tracy–Widom statistics, and secondly that the supervised analysis helps in interpretation of the recovered axes of variation.

The conventional statistic here is an ANOVA F-statistic. (See for instance [29]). We have here a “one-way layout,” where we want to test if the group means significantly differ. This amounts to a check on our Tracy–Widom statistic, which we compute ignoring the labels. We also routinely compute an F-statistic for every pair of populations, and each eigenvector (unpublished data). We give three examples of ANOVA analysis on real data. In the first, we look at population data from sub-Saharan Africa, genotyped with 783 microsatellites and 210 biallelic indels in the CEPH–HGDP dataset [30,31]. We group the West African and Bantu speaking populations (Yoruba, Bantu South Africa, and Bantu Kenya) as “Bantu” and also examine samples from San and Mandenka. We show plots of the first two eigenvectors in Figure 4. Table 1 shows the key statistics for this dataset. In Table 1, the ANOVA *p*-value is obtained from the usual *F*-statistic, and we apply ANOVA to each of the first three eigenvectors.

**Figure 4:**
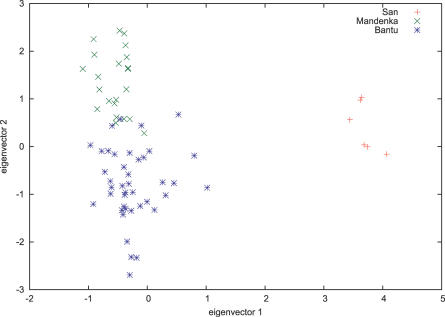
Three African Populations Plots of the first two eigenvectors for some African populations in the CEPH–HGDP dataset [30]. Yoruba and Bantu-speaking populations are genetically quite close and were grouped together. The Mandenka are a West African group speaking a language in the Mande family [15, p. 182]. The eigenanalysis fails to find structure in the Bantu populations, but separation between the Bantu and Mandenka with the second eigenvector is apparent.

**Table 1:**
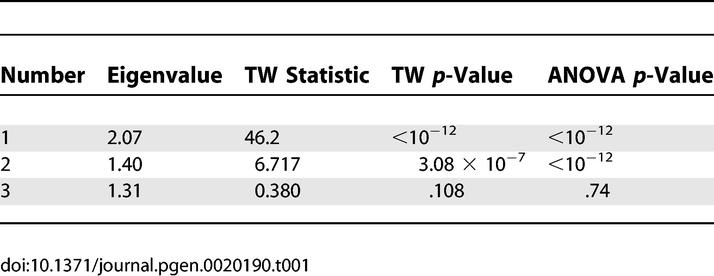
Statistics from HGDP African Data

There is excellent agreement between the supervised and unsupervised analysis. The lack of significance of the third eigenvector is an indication that no additional structure was apparent within the Bantu.

Our second study took samples from three regions: Northern Thailand, China (Han Chinese), and Japan. The last two population samples were available from the International Hapmap Project [32]. The Thai samples (25 individuals after removing some close relatives and outliers) were collected by J. Seidman and S. Sangwatanaroj as part of a disease study, though here we focus on the population genetics. Our analysis of these data used 40,560 SNPs.

In Figure 5 we plot the first two eigenvectors. Notice that the population separation is clear, but that the natural separation axes are not the eigenvectors. Further, the Thai and Chinese populations appear to show a cline, rather than two discrete clusters grouped around a central point. We suspect that this shows some evidence of genetic admixture, perhaps involving a population in Thailand that is related to the Chinese. (See also Figure 8, which we describe later.) Table 2 shows the eigenvalues, the TW significance, and an ANOVA *p*-value for the first three eigenvectors. Again there is excellent agreement between the supervised and unsupervised analyses.

**Figure 5:**
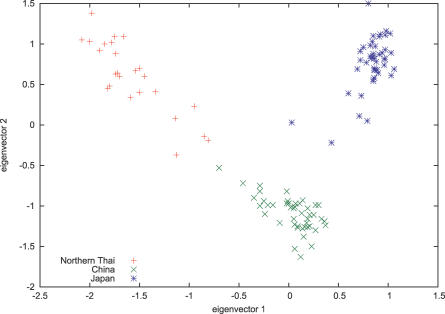
Three East Asian Populations Plots of the first two eigenvectors for a population from Thailand and Chinese and Japanese populations from the International Haplotype Map [32]. The Japanese population is clearly distinguished (though not by either eigenvector separately). The large dispersal of the Thai population, along a line where the Chinese are at an extreme, suggests some gene flow of a Chinese-related population into Thailand. Note the similarity to the simulated data of Figure 8.

**Table 2:**
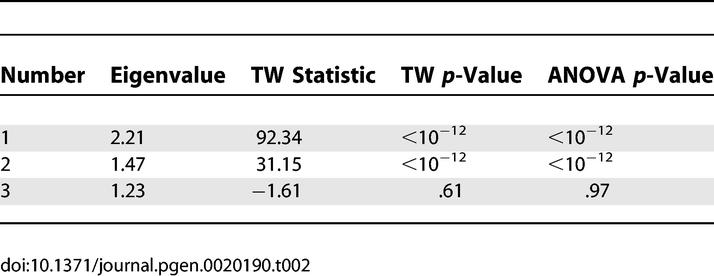
Statistics from Thai/Chinese/Japanese Data

In the third dataset, which was created and analyzed by Mark Shriver and colleagues [5], we have data from 12 populations. The missing data pattern showed some evidence of population structure, with the missing data concentrated in particular samples, populations, and SNPs. For this reason, we only used markers for analysis for which there was no missing data, and we corrected for LD using our regression technique (see below). The details of the data preprocessing steps are described in Methods. We analyzed samples from 189 individuals on 2,790 SNPs. On this dataset, we find the leading eigenvalue statistics to be as shown in Table 3.

**Table 3:**
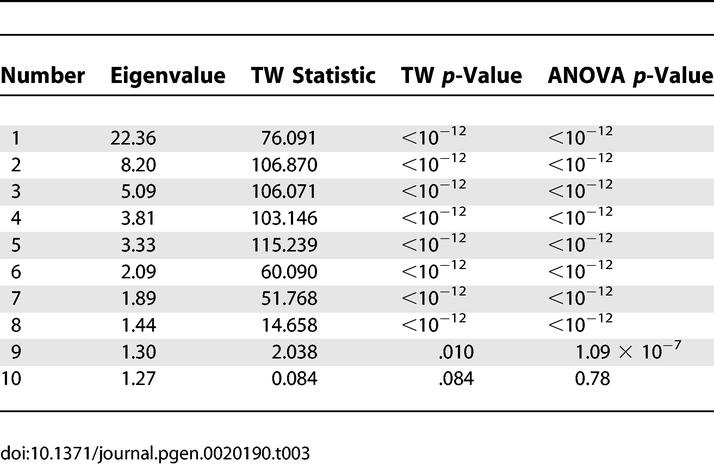
Statistics from Shriver Dataset

In all the datasets mentioned above, we have very good agreement between the significance of the TW statistic, which does not use the population labels, and the ANOVA, which does. This verifies that the TW analysis is correctly labeling the eigenvectors as to whether they are reflecting real population structure.

Shriver and colleagues [5], using different principal components methods and broken stick statistical analysis [27,28], recovered four significant components on this dataset. Our analysis has clearly recovered more meaningful structure, providing empirical validation of the power of this approach.

### An Estimate for the Data Size Needed for Significance

A recent paper by Baik, Ben Arous, and Péché [16] gives theorems for the asymptotics of the distribution of the largest eigenvalue of a sample covariance matrix when the true covariance matrix has a few eigenvalues greater than 1 and the rest equal to 1. This is the situation in genetic data for which there are just a few meaningful axes of variation. Unfortunately the theorems proved are only for the case of data matrices whose entries are complex numbers, but Baik, Ben Arous, and Péché conjecture that the results hold for real data, too. We state a form of the conjecture, which we call the BBP conjecture, and then provide evidence for its applicability to genetics.

Let *l*_1_ be the lead eigenvalue of the *theoretical* covariance matrix, with the remainder of the eigenvalues 1. Set *γ* ^2^ = *n*/*m*. Let *L*_1_ be the largest eigenvalue of the sample covariance. We will let *n*, *m* become large with the ratio *n*/*m* tending to a limit.

*BBP Conjecture* [16]:

(1) If l_1_ < 1 + 1/*γ, then as m,n → ∞*, *L*_1_, *suitably normalized, tends in distribution to the same distribution as when l*_1_ *= l.*

(2) If l_1_ > 1 + 1/*γ, then as m,n → ∞*, *the TW statistic becomes unbounded almost surely.*

That is, the behavior of *L*_1_ is qualitatively different depending on whether *l*_1_ is greater or less than 1 + 1/*γ*. This is a *phase-change* phenomenon, and we will define

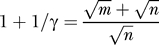
as the *BBP threshold*. This is an asymptotic result, showing that as the data size goes to infinity, the transition of the behavior, as *l*_1_ varies, becomes arbitrarily sharp. The result, as stated above, is proved in [16] for data where the matrix entries are complex numbers, and statement (2) of the conjecture is proved in [17], which demonstrates that the behavior is qualitatively different according to whether *l*_1_ is greater or less than 1 + 1/ *γ*. There seems little doubt as to the truth of statement (1) above. It has been shown (D. Paul, Asymptotic behavior of the leading sample eigenvalues for a spiked covariance model, http://anson.ucdavis.edu/~debashis/techrep/eigenlimit.pdf) that, under the assumptions of statement (1) above, the lead eigenvector of the sample covariance is asymptotically uncorrelated with the lead eigenvector of the theoretical covariance, but we believe that the question of the distribution of the leading eigenvalue is still open.

Consider an example of two samples each of size *m*/2, diverged from each other at time *τ,* where unit time is 2*N* generations, and assume that *N* is the effective population size. We assume *τ* is small, from which it follows that

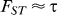
We find that

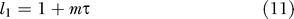
It follows that the BBP threshold is reached when

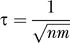



This is interesting by itself:

*Define D, the data size, to be the product of the number of samples and number of SNPs genotyped. For two subpopulations of equal sample size, the phase change threshold is reached when* 1/*F_ST_ is equal to the square root of the data size D, independently of the number of individuals and markers, at least when both are large.*

At a fixed data size, the expected value of the leading eigenvalue of the data matrix (and the power to detect structure) of course is a continuous function of *F_ST_,* but the BBP conjecture suggests that for large data sizes there will only be a small transition region. Above the region, detection of structure will be easy, and below it, impossible.

Let us take *nm* = 2^20^ (about one million genotypes), so that the BBP threshold is *F_ST_* = 2^−10^. We let *m* = 2*^k^* (*k* = 5…8) and set *n* = 2^20−*k*^ so that *nm* = 2^20^.

Now for each value of *m,* generate simulated data, varying *F_ST_* from 2^−13^ to 2^−7^. For each simulation, we compute *L*_1_, the TW statistic, and a *p*-value. We show the TW statistics in Figure 6.

**Figure 6:**
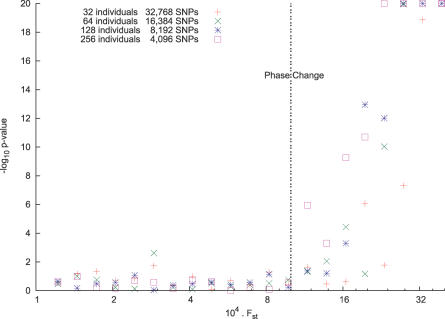
The BBP Phase Change We ran a series of simulations, varying the sample size *m* and number of markers *n* but keeping the product at *mn* = 2^20^. Thus the predicted phase change threshold is *F_ST_* = 2^−10^. We vary *F_S_* and plot the log *p*-value of the Tracy–Widom statistic. (We clipped −log_10_ *p* at 20.) Note that below the threshold there is no statistical significance, while above threshold, we tend to get enormous significance.

The phase change is evident. Further, from [16, p. 1650ff] (also see [17, Equation 1.10]): above the BBP threshold we have that

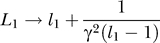
in probability as *m*,*n* → ∞. It then follows that above the BBP threshold, we can expect the TW statistic to be increasing with the number of individuals *m* if the data size *mn* is fixed. That is, increasing sample size, rather than marker number, is advantageous for detecting structure above the BBP threshold, but not below it. This effect is clearly visible in Figure 6 (note the behavior of the *p*-value for *m* = 256). We summarize:

*For two equal size subpopulations, there is a threshold value of F_ST_,* 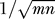 *, below which there will be essentially no evidence of population structure. Above the threshold, the evidence accumulates very rapidly, as we increase the divergence or the data size. Above the threshold for fixed data size mn, the evidence is stronger as we increase m, as long as n* ≫ *m.*

Another implication is that these methods are sensitive. For example, given a 100,000 marker array and a sample size of 1,000, then the BBP threshold for two equal subpopulations, each of size 500, is *F_ST_* = .0001. An *F_ST_* value of .001 will thus be trivial to detect. To put this into context, we note that a typical value of *F_ST_* between human populations in Northern and Southern Europe is about .006 [15]. Thus, we predict: *most large genetic datasets with human data will show some detectable population structure.*

The BBP phase change is *not* just a phenomenon of the eigenvector-based analysis we are discussing here. We suspect that at least for biallelic unlinked markers, no methods for detecting structure will do much better than our TW-based techniques. This implies that no method will have any significant success rate if population divergence is below the BBP threshold, while above threshold, reasonable methods will succeed. To test this we made a series of simulations, each with 1,600 biallelic markers and two populations each of size 50. We varied *F_ST_* and ran both our eigenanalysis and STRUCTURE. (See Methods for more detail about the simulations and analysis.) We were not successful in using STRUCTURE to produce a higher likelihood for the existence of two clusters rather than one except for the very largest *F_ST_* levels. We wanted to place our methods and STRUCTURE on a “level playing field.” Our PCA methods return a leading eigenvector, while running STRUCTURE with *K* = 2 clusters, returns for each individual the probability of belonging to cluster 1. We used a nonparametric idea, applying a probit transform to both the output of both the PCA and of STRUCTURE, and then running an ANOVA analysis, both for PCA and STRUCTURE output. (The probit transform uses order statistics (ranks) to map the observations into points appropriate if the underlying distribution is standard normal. See, for example, [33].) This amounts to carrying out an unsupervised analysis and then checking to see if the recovered “structure” reflects the truth.

Thus, we will compute three *p*-values: 1) a TW statistic from an unsupervised analysis; 2) an ANOVA *p*-value (F-statistic) after probit transform of the leading principal component; 3) an ANOVA *p*-value (F-statistic) after probit transform of the STRUCTURE cluster probabilities.

Table 4 shows the results from a representative set of runs: we show the geometric mean of the *p*-value in simulations, based on a TW statistic (unsupervised) or a nonparametric ANOVA analysis, both for the eigenanalysis and for STRUCTURE.

**Table 4:**
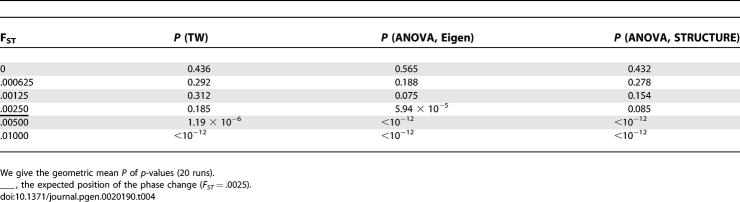
BBP Phase Change: Eigenanalysis and STRUCTURE

Here the BBP threshold is .0025. Below the threshold nothing interesting is found by the TW unsupervised statistic. Above the threshold, the TW statistic is usually highly significant, and the ANOVA analyses show that the true structure has become apparent. At the threshold we *sometimes* have recovered significant structure, but it will be hard (usually impossible) to tell if the structure is real or a statistical artifact. Below the threshold, the structure is too weak to be useful. In these runs, at the critical threshold, the eigenanalysis slightly outperformed STRUCTURE. We have not carefully investigated whether we could obtain better results by varying the STRUCTURE parameters.

Summarizing: below the threshold, neither procedure succeeds with reasonable probability, at the threshold success is variable, and above the threshold success is nearly guaranteed.

### Admixture

In an admixed population, the expected allele frequency of an individual is a linear mix of the frequencies in the parental populations. Unless the admixture is ancient—in which case the PCA methods will fail as everyone will have the same ancestry proportion—then the mixing weights will vary by individual. Because of the linearity, admixture does not change the axes of variation, or, more exactly, the number of “large” eigenvalues of the covariance is unchanged by adding admixed individuals, if the parental populations are already sampled. Thus, for example, if there are two founding populations, admixed individuals will have coordinates along a line joining the centers of the founding populations.

We generated simulated data, by taking a trifurcation between populations (*A*,*B*,*D*) 100 generations ago. Population *C* is a recent admixture of *A* and *B*. The mixing proportion of *A* in an individual from C is Beta-distributed *B*(3.5,1.5) so that the average contribution of population *A* in an individual of population *C* is .7 (see Figure 7). Effective population sizes are 10,000 for each population. We then simulated data for 10,000 unlinked markers (more details are in the Methods section). *F_ST_* between any pair of *A*,*B*,*D* is .005. We are attempting to mimic the data of Figure 5, and chose to run our analysis on simulated samples from populations *B,C,D,* not using samples from *A*. We expect two significant eigenvalues corresponding to the splits of populations *B,C,* and *D*. If population *A* is included in the analysis, we also get just two significant eigenvalues, as predicted by theory. This is what is observed (unpublished data), with, as predicted, the admixed population not adding to the number of axes of variation (the third eigenvalue is not significant). In Figure 8 we show a plot of the first two eigenvectors. Note the dispersion of population *C* along a line. This is diagnostic of admixture. The resemblance of Figures 5 and 8 is striking.

**Figure 7:**
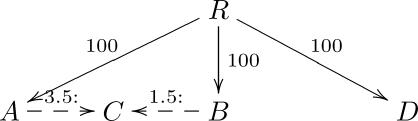
Simulation of an Admixed Population We show a simple demography generating an admixed population. Populations *A,B,D* trifurcated 100 generations ago, while population *C* is a recent admixture of *A* and *B*. Admixture weights for the proportion of population *A* in population *C* are Beta-distributed with parameters (3.5,1.5). Effective population sizes are 10,000.

**Figure 8:**
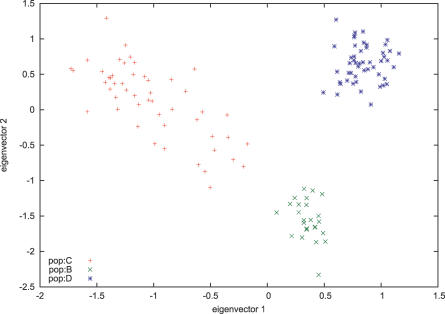
A Plot of a Simulation Involving Admixture (See Main Text for Details) We plot the first two principal components. Population C is a recent admixture of two populations, B and a population not sampled. Note the large dispersion of population C along a line joining the two parental populations. Note the similarity of the simulated data to the real data of Figure 5.

There remain issues to resolve here. Firstly, recent admixture generates large-scale LD which may cause difficulties in a dense dataset as the allele distributions are not independent. These effects may be hard to alleviate with our simple LD correction described below. STRUCTURE [10] allows careful modeling. Secondly, more ancient admixture, especially if the admixed population is genetically now homogeneous, may lead to a causal eigenvalue not very different from the values generated by the sampling noise. Suppose, for example, in our simulation above, we let population *C* mate panmictically for another 20 generations. Then we will get three clusters for *A, B, C* that are nearly collinear, but not exactly because of the recent 20-generation divergence, which is reflecting genetic drift unique to that population.

A third issue is that our methods require that divergence is small, and that allele frequencies are divergent primarily because of drift. We attempted to apply our methods to an African-American dataset genotyped on a panel of ancestry-informative markers [34]. The Tracy–Widom theory breaks down here with dozens of “significant” axes that we do not believe have genetic meaning. Perhaps this is to be expected, as on our informative panel *F_ST_* is big (.58) and the theory could be expected to perform poorly. In addition our methods are here not dealing adequately with LD caused by large admixture blocks.

This is an issue for our TW techniques, but not for PCA as such. Indeed, on this dataset the correlation of our principal eigenvector with the estimated European ancestry for each individual recovered by the admixture analysis program ANCESTRYMAP [12] is a remarkable .995 (STRUCTURE produces similar results). ANCESTRYMAP has complex modeling of admixture LD, and was also provided with parental allele frequencies, but did no better than the simple PCA. (There is an issue of interpretation here: the leading eigenvector is almost perfectly correlated with ancestry, but to infer actual ancestry proportions an affine transform must be applied, translating and scaling the values. In practice, some parental allele frequencies will be needed to determine the appropriate transform. A similar issue arises with STRUCTURE if parental frequencies are unknown.)

Finally, if “admixture LD” is present, so that in admixed individuals long segments of the genome originate from one founder population, simple PCA methods will not be as powerful as programs such as STRUCTURE [10], ADMIXMAP [11], and ANCESTRYMAP [12], where there is careful modeling of the admixture blocks and the transitions. The power of these methods lies in the fact that genome-wide samples may have similar proportions of inheritance from the ancestral populations, but locally they will inherit either 0, 1, or 2 alleles from each ancestral population. Methods that specifically attempt to assign local ancestries will be able to determine the specific patterns typical of each ancestral population locally. An interesting and challenging problem is to build tools that retain the power of these more complex models on admixed data and that also run rapidly on large datasets.

### Correcting for LD

The theory above works well if the markers are independent (that is have no LD), but in practice, and especially with the large genotype arrays that are beginning to be available, this is difficult to ensure. In extreme cases uncorrected LD will seriously distort the eigenvector/eigenvalue structure, making results difficult to interpret. Suppose, for example, that there is a large “block” [35,36] in which markers are in complete LD, and we have genotyped many markers in the block. A large eigenvector of our Wishart matrix *X* will tend to correlate with the genotype pattern in the block (all markers producing the same pattern). This will distort the eigenvector structure and also the distribution of eigenvalues.

We recommend the following if LD between markers is a concern in the data. Pick a small integer *k* > 0, corresponding to the number of adjacent markers one uses for adjustment (*k* = 1 will often suffice). In the data matrix M we will “predict” each column by running a multivariate regression on the *k* previous columns. We then will analyze the residuals. Concretely: we first form *M,* as in Equation 2. For each column *j*

Set:

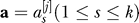


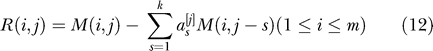
Choose **a** to minimize

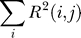
and now calculate *X* = *RR′* instead of *MM′*. It is first important to check that in the absence of LD the suggested correction does not seriously distort the Tracy–Widom statistic. In Figure 9A and 9B we show P–P plots, uncorrected, and with five levels (*k* = 1…5) of correction. The first figure is with 100 individuals and 5,000 markers, the second with 200 individuals and 50,000 markers. Then in Figure 10A and 10B we analyze a simulated dataset with severe LD. We generate blocks in perfect LD, in which the probability that a block contains *L* markers is 2^−L^. We show the corresponding plots. Note that here the uncorrected statistic is distributed quite differently than the Tracy–Widom distribution. Our suggested correction strategy seems to work well, and should be adequate in practice, especially as most large genotype arrays will attempt to avoid high levels of LD. We would recommend that before analyzing a very large dataset with dense genotyping, one should filter the data by removing a marker from every pair of markers that are in tight LD.

**Figure 9:**
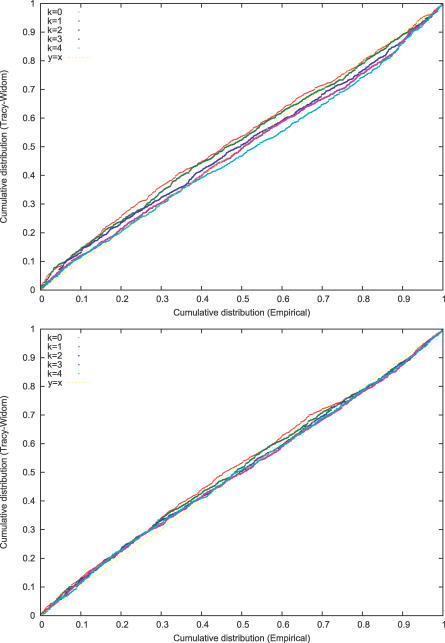
LD Correction with no LD Present P–P plots of the TW statistic, when no LD is present and after varying levels (*k*) of our LD correction. We first show this (A) for *m* = 500, *n* = 5,000, and then (B) for *m* = 200, *n* = 50,000. In both cases the LD correction makes little difference to the fit.

**Figure 10:**
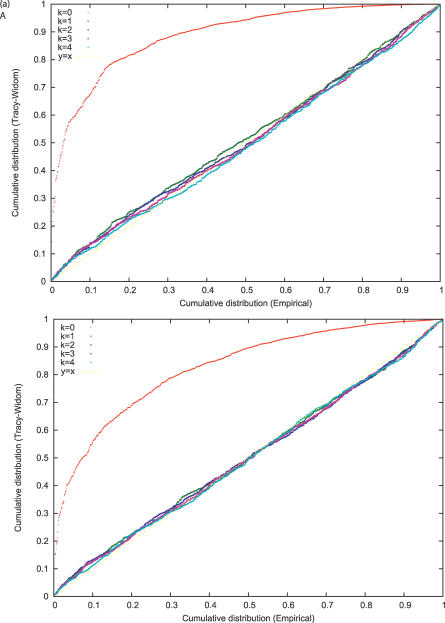
LD Correction with Strong LD (A) Shows P–P plots of the TW statistic (*m* = 100, *n* = 5,000) with large blocks of complete LD. Uncorrected, the TW statistic is hopelessly poor, but after correction the fit is again good. Here, we show 1,000 runs with the same data size parameters as in Figure 2A, *m* = 500, *n* = 5,000, varying *k,* the number of columns used to “correct” for LD. The fit is adequate for any nonzero value of *k*. (B) Shows a similar analysis with *m* = 200, *n* = 50,000.

### Comparison with STRUCTURE

In the work above on the BBP phase change, we already showed some comparisons between STRUCTURE and our methods. A fair comparison to STRUCTURE is not easy, as the two programs have subtly different purposes and outputs. STRUCTURE attempts to describe the population structure by probabilistic assignment to classes, and we are attempting to determine the statistically significant “axes of variation,” which does not necessarily mean the same thing as assigning individuals to classes.

Our impression, confirmed by Table 4, is that when our analysis finds overwhelmingly evident population structure, then STRUCTURE will as well, and when nothing at all is found, STRUCTURE will fail, too. In a problem where the effect is marginal, it may be hard to say which analysis is preferable.

STRUCTURE is a sophisticated program with many features we have not attempted to match. STRUCTURE has an explicit probability model for the data, and this allows extra options and flexibility. It incorporates a range of options for ancestry and for allele frequencies, and has explicit options for modeling microsatellite distributions.

On the other hand, eigenanalysis has advantages over STRUCTURE. First, it is fast and simple, and second, it provides a formal test for the number of significant axes of variation.

One future possibility is to somehow incorporate recovered significant eigenvectors into STRUCTURE—in particular with regard to choosing the number of subpopulations, which is not statistically robust in the STRUCTURE framework. A sensible default for the number of clusters in STRUCTURE is one more than the number of significant eigenvalues under the TW statistic.

### Missing Data and Other Problems

The most problematic issue when applying any method to infer population structure is that genotyping may introduce artifacts that induce apparent (but fallacious) population structure.

Missing genotypes by themselves are not the most serious concern. Simply setting *M*(*i*,*j*) = 0 in Equation 3 if marker *j* is missing for individual *i* is reasonable if we are testing the null, that there is no structure, and the missing data is “missing at random.” Unfortunately “informative missingness” [37,38] is extremely frequent in genetic data. Probably the most common and serious issue is that with current technology, heterozygotes are more difficult to call than homozygotes. Thus, true heterozygotes are more likely to be called as missing. This is discussed in detail in [38], which is recommended as a very useful discussion of the issues, especially as they apply to medical genetics. If DNA quality (or quantity) varies among our samples, then certain individuals may have an unusual amount of missing data, and then appear as outliers in our eigenanalysis—we in fact have seen this in many runs on real data.

Another issue that may produce confounding effects is if data from different populations or geographical areas is handled differently (which may be inevitable, especially in the initial processing); then, in principle, this may induce artifacts that mimic real population structural differences. Even restricting analysis to markers with no missing data, apart from an inevitable power loss, does not necessarily eliminate the problems. After all, if a subset (the missing data) is chosen in a biased way, then the complementary subset must also be biased.

We have no complete solution to these issues, though there is no reason to think that our eigenvector-based methods are more sensitive to the problems than other techniques [9]. One check we do recommend is to generate a test matrix by taking the initial counts *C*(*i*,*j*) to be 0 if the corresponding data is present; otherwise, set *C*(*i*,*j*) = 1. This is equivalent to only focusing on the pattern of missing data. The eigenanalysis on this test matrix will show significant TW statistics if the missing data *by itself* is showing evidence of population structure. If so, the results should be regarded with some suspicion, especially if the eigenvectors show high correlation to the eigenvectors of the main analysis. We here echo [38] and recommend that the analyst should “control all aspects of source, preparation and genotyping, using the paradigms of blindness and randomization,” but, as the reference states, this will not always be possible.

Another possible source of error, where the analyst must be careful, is the inclusion of groups of samples that are closely related. Such a “family” will introduce (quite correctly from an algorithmic point of view) population structure of little genetic relevance, and may confound features of the data of real scientific interest. We found that this occurred in several real datasets that we analyzed with eigenanalysis and in which related individuals were not removed.

## Discussion

For many genetic datasets, it is important to try to understand the population structure implied by the data. STRUCTURE [9], since its introduction, has been the tool of choice, especially for small datasets. We think we have provided some evidence that PCA has advantages also, as it is fast, easily implemented, and allows accurate testing of significance of a natural null model.

We can only uncover structure in the samples being analyzed. As pointed out in [39], the sampling strategy can affect the apparent structure. Rosenberg et al. [29] give a detailed discussion of the issue, and of the question of whether clines or clusters are a better description of human genetic variation. However, our “axes of variation” are likely to be relatively robust to this cline/cluster controversy. If there is a genetic cline running across a continent, and we sample two populations at the extremes, then it will appear to the analyst that the two populations form two discrete clusters. However, if the sampling strategy had been more geographically uniform, the cline would be apparent. Nevertheless, the eigenvector reflecting the cline could be expected to be very similar in both cases.

Our methods are conceptually simple, and provide great power, especially on large datasets. We believe they will prove useful both in medical genetics, where population structure may cause spurious disease associations [1,40–43]; and in population genetics, where our statistical methods provide a strong indication of how many axes of variation are meaningful. A parallel paper [14] explores applications to medical genetics.

### Mathematical Details

#### A moments estimator.

We justify our estimator of the “effective number of markers.”

Theorem 1.

*Let λ*_1_, *λ*_2_,… *λ_m_ be eigenvalues of an m × m Wishart matrix MM′, where M is m × n with entries that are Gaussian with mean* 0 *variance σ* ^2^ *. Define*

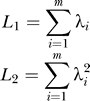



*If n,σ* ^2^ *are unknown, estimates are:*

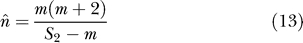


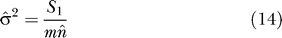

*where*

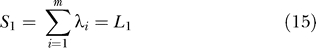


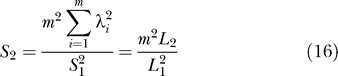



*With these values of* *n^* *and* 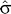 *, the observed values of L*_1_ *and* 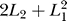 *are equal to their expected values.*

Note that in this section we define our Wishart as *MM′*, *not* 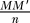 , as *n* is unknown. This scaling hardly matters in applications, as our procedures are always scale-invariant. That is, we avoid assumptions on the variance of the Gaussian entries of *M*.

Proof:

Let **a** = (*a*_1_,*a*_2_,…*a_m_*) be a random vector uniformly distributed on the unit *m*-sphere. 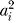 is Beta (1/2,(*m* − 1)/2)-distributed, and it follows that

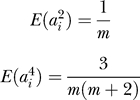
Let 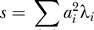 . Then

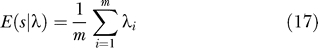


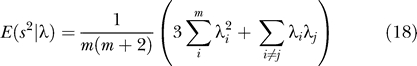


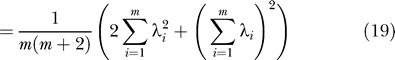



To obtain the distribution of *s,* unconditioned on *λ,* we see that we can write *s* as

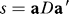
where *D* = *diag*(*λ*_1_, *λ*_2_,… *λ_m_*). After an orthogonal transformation

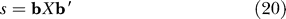
where *X* is our Wishart matrix and **b** is uniform (isotropic) on the unit sphere. By properties of the Gaussian distribution, the distribution of *s* as given by Equation 20 is independent of **b**. We choose **b** to be (1,0,0,…). It follows that *s*/*σ* ^2^ is distributed as a 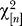 variate so that *s* = 2*σ* ^2^ *G* where *G* is Γ(*n*/2)-distributed. Thus,

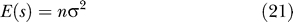


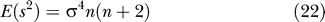
Comparing Equations 17 and 21, this proves:

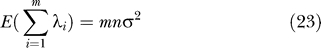
and comparing Equations 19 and 22, we find:

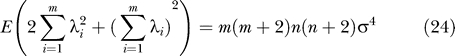
From Equations 23 and 24:

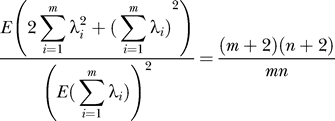
so that a natural estimator for *n* is:

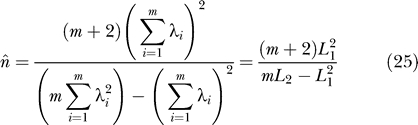
We then obtain as an estimate for *σ*:

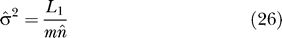
If we set:

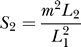
then Equation 25 simplifies to:

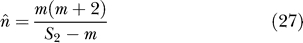
This completes the proof of Theorem 1.

It would be interesting to estimate the standard error for *n^* .

We next show that normalizing the eigenvalues of an *m* × *m* Wishart to sum to *m* does not change the asymptotics of the largest eigenvalue. In our data analysis we always normalize the empirical eigenvalues in this way.

Theorem 2.

*Consider a Wishart matrix X with eigenvalues λ_i_, originating from an m × m matrix M whose entries are Gaussian with mean* 0 *and variance* 1*. That is,* 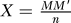 *. Let λ*_1_ *be the largest eigenvalue of X. Define*

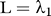


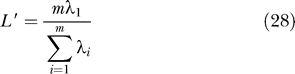

*Define τ by*

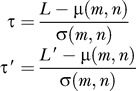

*which normalizes L,L′ by the Johnstone normalization of Equation 7 with μ and σ defined as in Equations 5 and 6. Then L and L′ both tend in distribution to the Tracy–Widom distribution as m*,*n → ∞,n/m → γ > 1. That is, the normalization of Equation 28 does not change the asymptotic distribution of L.*

Proof:

Let

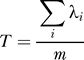
Then

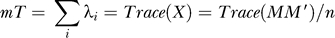
So

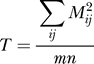
Each entry of *M* is standard normal, and so *T* has mean 1 and standard deviation

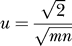
Let *s* = *σ*(*m*,*n*) be the scale factor of the Johnstone normalization. Then we can show (we used Maple) that as *n* → ∞,

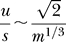
Write *T* = 1 + *x* so that *x* has mean 0 and standard deviation *u*. Thus, *x*/*s* → 0 in probability as *m* → ∞.

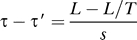


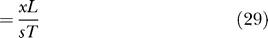



We now show that this implies that *τ* − *τ*′ tends to 0 in probability. From the definition of *μ*(*m*,*n*) in Equation 5, we have *μ*(*m*,*n*) < 4. Pick a constant (say 10) >4. Since as *m* → ∞, (*L* − *μ*(*m*,*n*))/*σ*(*m*,*n*) tends to TW in distribution, and *σ*(*m*,*n*) → 0, it follows that *P*(*L* > 10) tends to 0 as *m* → ∞. Similarly, *P*(*T* < 1/2) tends to 0. Take *ɛ* > 0. From Equation 29:

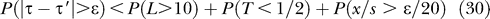



All three probabilities on the right hand side of Equation 30 can be made arbitrarily small for large enough *m*.

By Johnstone's theorem, *τ* → TW in distribution, and so *τ′* → TW also.

### The Spectrum of the Covariance Matrix

We now turn to genetic (genotype count) data, and analyze the theoretical covariance matrix of the data. We concentrate on the covariance of the sample genotypes at a single biallelic marker. Note that in contrast to the results for a Wishart discussed in Theorem 2, we are now interested in a case where there is population structure, which implies dependence between the samples.

Consider sampling a marker from samples belonging to *K* populations. Suppose the allele has frequency *p_i_* in population *i*. We sample diploid genotypes, obtaining counts *C_j_* of the variant allele from sample *j*. We suppose sample *j* belongs to population *i* = *i*(*j*), and that the sample size for population *i* is *M*(*i*). We discuss the spectrum (eigenvalues) of the covariance matrix of the raw counts *C_j_*. Note that this is for the *theoretical* covariance not the *sample* covariance.

We must specify the covariance of the population frequency vector

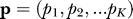



We assume that there is a hidden allele frequency *P* whose exact distribution will not be important to us, but is diffuse across the unit interval (0,1). Then conditional on *P* we assume that **p** has mean *P*(1,1,…,1) and covariance matrix *P*(1 – *P*)*B* where *B* is independent of *P*. This is a natural framework, used (filling in details variously) by Balding and Nichols [44], Nicholson et al. [18], and STRUCTURE [9] in the correlated allele mode. For small population divergence, we can take the diagonal entry *B_ii_* as the divergence (*F_ST_*) between *P* and *p_i_*. Set

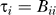
and assume that all *τ_i_* are of order *τ,* which is small. Conditional on **p**, then the *C_j_* are independent. *C_j_* has mean *p* and variance 2*p*(1 − *p*) where *p* = *p_i_*_(*j*)_. This assumes Hardy–Weinberg equilibrium in each of the *K* populations.

Theorem 3.

*With the assumptions above, define*

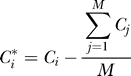

so that C_i_^*^ has mean 0. Let V^*^ be the covariance matrix of C^*^ and set 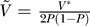 . *Conditional on the root frequency P:*

1. 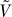 does not depend on *P*.

2. 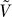 has an eigenvalue 0 with eigenvector 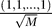 .

3. 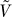 has for each *k* (1 ≤ *k* ≤ *K*), *M*(*k*) − 1 eigenvalues equal to 1 − *τ_k_*. (We will call these the *small* eigenvalues.)

4. 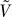 has *K* − 1 eigenvectors that span a vector space F^*^ consisting of vectors **v** of length *M* whose coordinates are constant on samples from each population, and such that the sum of the coordinates of **v** are 0.

5. If the matrix *B* (the scaled covariance of the population frequencies **p**) has rank *r,* then *r* − 1 of the eigenvalues of 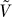 that correspond to eigenvectors in F^*^ depend on *B*. (So if *B* has full rank, all these eigenvalues depend on *B.*) If we allow each sample size *M*(*k*) → ∞, then then all such eigenvalues also → ∞. (We will call the corresponding eigenvalues the *large* eigenvalues).

Proof:

Let *V* be the covariance matrix of the counts *C*. Regard *V* = || *V_ij_* || as a linear operator in the natural way. Write *π*(*i*) for the population index of sample *i*(1 ≤ *i* ≤ *K*). We can write *V* = *V_ij_* as *D* + *W* where *D* is a diagonal matrix with the diagonal element *D_ii_* = *d_π_*_(*i*)_ and *W_ij_* = *q_π_*_(*i*)*,π*(*j*)_. So the covariance structure depends only on the population labels of the samples. It follows that the vector space of *M* long column vectors has an orthogonal decomposition into subspaces invariant under *V* consisting of: 1) a subspace *F* of vectors whose coordinates are constant within a population. *F* has dimension *K*; 2) subspaces *S_i_* (1 ≤ *i* ≤ *K*). Vectors of *S_i_* are zero on samples not belonging to population *i,* and have coordinate sum 0, which implies that they are orthogonal to *F*. It now follows that *V* has *K* eigenvectors in *F,* and for each *k* (1 ≤ *i* ≤ *K*), (*M*(*k*) − 1 eigenvectors in *S_k_* each of which have the same eigenvalue *λ_k_*. Conditional on **p**, *V* acts on *S_k_* as 2*p_k_*(1 − *p_k_*)*I* where *I* is the identity matrix. (The factor 2 comes from the two chromosomes sampled for each individual.) Thus,

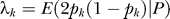
Now

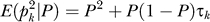
and so the eigenvalues corresponding to eigenvectors of *S_k_* are:

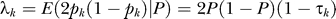



V^*^ and *V* act identically on *S_k_,* the vectors of which have coordinate sum 0, so this proves assertion 3 of Theorem 3.

We now consider the action of *V* on the *K*-dimensional subspace *F*. It is convenient to define 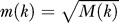 , a quantity we will need repeatedly. Let for each *k* (1 ≤ *k* ≤ *K*), **f** ^[*k*]^ be the vector whose coordinates are 0 except for samples *i* where *π*(*i*) = *k,* and where for such samples:

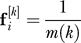



The vectors **f** ^[*k*]^ form an orthonormal basis for *F*. Write *d_k_* = **f** ^[*k*]^.*C*. Set *E* to be the diagonal matrix

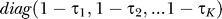
It is easy to calculate that the random variables *d_k_* have, conditional on *P,* covariance matrix *R,* where

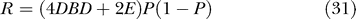
and *D* = *diag*(*m*(1),*m*(2),…*m*(*K*)). Here *E* corresponds to sampling noise.

In the main paper we subtract the sample mean from the counts *C*. So define the *M* long vector **1** = (1,1,…,1). Then

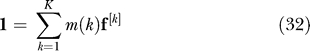



Set 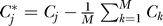 , this is a linear transform *T* where

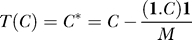
We are interested in the action of *T* on *F*. Write

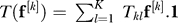
 Then from Equation 32, regarding *T* as a *K* × *K* matrix (abusing notation): *T* = *I* − *Q* where *I* is the identity matrix and

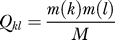



Set

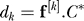
It now follows from Equation 31 that if R^*^ is the covariance matrix of the *d_k_,* then

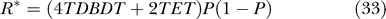



This is enough to prove that 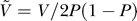 does not depend on *P* (assertion 1 of Theorem 3).

Next, *T*(**1**) – R^*^(**1**) = **0**, which proves assertion 2 of Theorem 3. The space F^*^ of vectors *F* orthogonal to 1 is invariant under *V* and 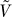 , thus R^*^ will have *K* − 1 eigenvectors of F^*^ (assertion 4 of Theorem 3). If *B* has rank *K* (which will be true except in special cases), then *TDBDT* has rank *K* − 1 and if *M*(*k*) → ∞ for each *k,* then R^*^ will have *K* − 1 nonzero eigenvalues which become arbitrarily large. More generally, if *B* has rank *r,* then the matrix *TDBDT* will have rank *r* − 1, and the *r* − 1 eigenvalues of R^*^ that depend on *B,* again will become arbitrarily large as *M*(*k*) → ∞. Note that the matrix *TET* which arises from sampling noise is bounded. (In fact *TET* is a contraction and has all eigenvalues less than 1.) This completes the proof of Theorem 3.

The case in which *B* does not have full rank occurs if there has been a genetically recent admixture between two or more populations. In this case, even if there are *K* clearly distinct populations, fewer than *K* − 1 eigenvalues will become large as the sample size increases.

### Definition of the TW Density

For completeness, we define the TW density. Our description is taken from [22].

Let *q*(*x*) be the solution of the differential equation:

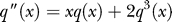
with the boundary condition:

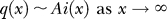
and *Ai*(*x*) is the Airy function. Then the TW distribution is given by:

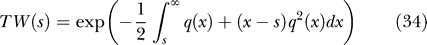



A table of the TW right-tail area, and density, is available on request.

### Some Questions in Theoretical Statistics

We believe this work raises some challenges to theoretical statisticians. Our results with genetic simulations would be even more convincing if there were theorems (say for the Wishart case where the data matrix has Gaussian entries) that showed: 1) that using the effective number of markers calculated by Equation 10 instead of the true number of markers does not affect the asymptotics; 2) that the BBP phase change holds for real Wishart matrices as well as for complex; 3) in Figures 2 and 3 the P–P plots show a noticeably better fit at the high end, corresponding to low *p*-values. Explain!

## Methods

### Datasets used.

For the data used in Figure 4, we use the H952 subset of the CEPH–HGDP panel [30,31,45] where some atypical samples and pairs of close relatives have been removed.

For the data used in Figure 5, we use an unpublished sample collected and genotyped by Dr. Jonathan Seidman and Dr. S. Sangwatanaroj. This consisted of 25 samples from Northern Thailand (after removing some individuals who are close relatives of people whose samples we retained) and 45 samples each from China and Japan (data drawn from the International Human Haplotype Map Project [32]). The Northern Thai samples were genotyped using an Affymetrix Xba chip. The dataset analyzed consisted of the overlap between the SNPs successfully genotyped in HapMap and the Affymetrix chip, and included 40,560 SNPs.

For the data of Mark Shriver and colleagues [5], we analyzed only autosomal data where no SNP had any missing data. We removed one individual who was a duplicate, two Burunge and Mbuti samples that represented close relatives of other samples, and nine Nasioi individuals who our data suggest are part of one or two extended families.

### Algorithm details.

In the eigenanalysis of the Shriver data, we examine no more than two markers as independent regression variables for each marker we analyze, insisting that any marker that enters the regression be within 100,000 bases of the marker being analyzed. This slightly sharpens the results. Varying these parameters made little difference.

For all STRUCTURE runs, we ran with a burn-in of 10,000 iterations with 20,000 follow-on iterations, and no admixture model was used. Computations were carried out on a cluster of Intel Xeon compute nodes, each node having a 3.06-GHz clock.

For our coalescent simulations, we assumed a phylogenetic tree on the populations, and at each simulated marker, ran the coalescent back in time to the root of the tree. At this point we have a set of ancestors *A* of the sampled chromosomes. We now assume that the marker is biallelic and that the population frequency *f* of the variant allele in the ancestral population is distributed uniformly on the unit interval. Sample the frequency *f* and then choose an allele for each ancestor of *A,* picking the allele for each ancestor with probability *f*. Now retain the marker if it is polymorphic in our samples. This process is mathematically equivalent to having a very large outgroup population diverging from the sampled populations at the phylogenetic root, with the population panmictic before any population divergence, and ascertaining by finding heterozygotes in the outgroup. If our simulated samples have *n* individuals, our procedure yields a sample frequency that is approximately uniform on (1,2,…,2*n* − 1).

For the admixture analysis that created the plot of Figure 8 we had a population *C* that was admixed with founder populations *A* and *B*. For each individual of *C,* we generated a mixing value *x* that is Beta-distributed B(3.5,1.5). Now for each marker independently, the individual was assigned to population *A* with probability *x* or *B* with probability 1 − *x*.

## Supporting Information

SMARTPCA, a software package for running eigenanalysis in a LINUX environment, is available at our laboratory: http://rd.plos.org/david_reich_laboratory.

## Abbreviations

LD: linkage disequilibrium
PCA: principal components analysis
